# Robust Trajectory and Resource Optimization in UAV-Enabled IoT Networks under Probabilistic LoS Channel in Presence of Jammers

**DOI:** 10.3390/s23010070

**Published:** 2022-12-21

**Authors:** Zhi Ji, Yufang Gao, Wendong Yang, Chuanzhen Rong

**Affiliations:** 1 The College of Communications Engineering, Army Engineering University of PLA, Nanjing 210007, China; 2 School of Electronic Science and Engineering, Nanjing University, Nanjing 210023, China

**Keywords:** probabilistic LoS channel, IoT devices, UAV communications, anti-jamming, robust design

## Abstract

This paper studies the anti-jamming problem of unmanned aerial vehicle (UAV)-enabled Internet of Things (IoT) communication networks in the presence of a jammer under the accurate probabilistic line-of-sight (LoS) model. Our goal is to maximize the information collection throughput of the system under the assumption that only the jammer’s approximate location is known. To this end, we formulate a throughput maximization problem by optimizing the UAV trajectory, the IoT devices’ transmit power, and communication scheduling under the accurate real-time probabilistic LoS channel. However, the proposed optimization problem is non-convex and coupled, and hence intractable to be solved. In order to tackle the problem, a robust iterative algorithm is proposed by leveraging the block coordinate descent (BCD) method, the successive convex approximation (SCA) technology, the difference of convex (D.C) programming approach, and the S-procedure. Extensive simulation results show that our proposed algorithm significantly improves the system throughput while achieving a practical anti-jamming effect compared with the benchmark algorithms.

## 1. Introduction

After nearly ten years of technological development and transformation, the Internet of Things (IoT) has unknowingly changed our lives. IoT refers to the interconnection of ubiquitous devices/facilities and intelligent devices via wireless/wired long-distance /short-distance management and services [1]. IoT applications involve all aspects of our lives, such as homes, transportation, industry, and medical care, making society more intelligent and efficient. It is expected that there will be billions or even trillions of IoT devices connected to the Internet in the future [2,3]. Due to the randomness and decentrality of the distribution of the IoT devices, the available energy and transmission range of partial wireless devices are very limited when they are used for environmental monitoring or other similar goals [4]. For example, the IoT devices in intelligent agriculture/cities are usually powered by batteries, required to transmit over distances of up to tens of kilometers, and are expected to last for more than ten years [4]. Therefore, it is still a challenge for many IoT devices to be interconnected and achieve massive data acquisition, storage, and transmission at the same time [5].

An attractive solution to the challenges noted above is introducing unmanned aerial vehicles (UAVs) into IoT systems [6,7,8]. In the UAV-enabled IoT systems, aiming at the challenge of the limited transmission distance of terrestrial wireless devices, a UAV can serve as an airborne relay to enhance the connectivity and extend the coverage range of the wireless networks. At the same time, it is convenient to realize services such as instant data collection, target identification, and temporary communication [9], and effectively reduce the communication overhead of the wireless equipment and prolong the life of the IoT system [9]. Therefore, UAV-enabled IoT systems can be more efficient and successful in completing many tasks requiring a high level of workforce, e.g., oilfield and high-voltage line inspection, and mine monitoring. Recently, many researchers have researched the promising technologies and applications of UAV-enabled IoT communications [10,11,12,13,14], showing that, after proper UAV trajectory design and corresponding communication resources optimization, the system performance can be significantly improved.

However, the open space of radio transmission makes wireless communication vulnerable to the interference of the external environment. For example, a portable shoulder-mounted rifle can use directional electromagnetic waves to completely invalidate the UAV GPS or WiFi signal, which results in the UAV needing to hover in place or land directly at the designated destination under the attacker’s control. When UAVs suffer from jamming, the communication link between the UAV and the ground control station is interrupted, making the UAV unable to complete the scheduled task. Electromagnetic jamming can be divided into two distinct types: malicious jamming and unintentional jamming. Specifically, malicious jamming is the behavior where a specific device outside the communication system is transmitting high-power signals to interfere with legal communications. In addition, the information about a malicious jammer is unknown and not controlled by the legitimate system, which is more harmful to the communication system and even causes legitimate communication interruption [15,16]. To combat jamming attacks, by adjusting the UAV’s position adaptively over time, one can achieve excellent safety and anti-jamming performance, and there are corresponding anti-jamming technologies when facing malicious jamming attacks [17,18,19,20,21]. Specifically, references [17,18] utilized convex optimization techniques to achieve secure transmission of UAV communications. With the help of cognitive radio and machine learning, the authors in [19] studied the dynamic spectrum anti-jamming problem. Literature [20] adopts the Bayesian Steinberg game method; by formulating the competitive relationship between the UAV (user) and the jammer, the anti-jamming transmission of the system is realized. The authors in [21] analyzed the spectrum sharing technology and cases in air–ground integrated networks and simultaneously discussed the robust technologies tackling uncertain and unknown information.

Due to the various communication equipment and protocols and the changeable environment, UAV communication is in a highly dynamic and uncertain physical and electromagnetic environment. No communication or control entity can grasp the system’s global information in real-time accurately. However, the current research on UAV communication generally supposes that the system’s state information is known or can be accurately predicted. Therefore, when optimizing or planning a UAV communication network, it is necessary to study the robustness system to ensure that the UAV communication system can still perform normally in an uncertain environment [22,23,24,25,26]. In this paper, we will devote ourselves to the design of a robust UAV communication system.

UAV trajectory design is critically dependent on the U2G (UAV to ground) channel modeling. To our knowledge, the majority of the existing work dedicated to the UAV communication’s anti-jamming design rarely paid attention to channel modeling. Specifically, in the offline design of UAV trajectory, the deterministic LoS channel following the free-space path loss model has been widely used in most of the existing work [27,28,29,30,31]. However, the highly dynamic nature of UAVs determines the real-time changing characteristics of the channel, and it is generally inaccurate to use a simplified channel model in UAV communication [32], especially in urban/suburb areas. Therefore, it is essential to explore more accurate channel modeling in UAV anti-jamming design.

Recently, two channel models, named Rician fading channel model dependent on elevation angle [33] and probabilistic LoS channel model [34] have been proposed, in which the probabilistic LoS channel model statistically characterizes the channel state by modeling the occurrence probability of LoS and non-line-of-sight (NLoS) and is expressed by the heuristic function of the U2G elevation angle. The authors in [35] achieved the maximization of the minimum rate by optimizing the ground nodes scheduling and UAVs’ 3D trajectories in the presence of a jammer. However, it was assumed that the jammer’s location information is fully known, which is usually impossible in practice. In the UAV communication network, the information of malicious jammer is difficult for us to grasp and use. Therefore, under the probabilistic LoS channel, the anti-jamming problem where the malicious jammer’s location information is imperfect needs to be explored and studied.

Based on the above observations, this paper proposes a joint robust optimization scheme under the probabilistic LoS channel model. In the presence of a jammer with imperfect location information, UAV collects information from the IoT devices. The IoT devices need to establish a reliable communication link to a legitimate UAV in the presence of the jammer. We model a more accurate U2G probabilistic LoS channel model and aim to maximize the collection throughput by optimizing the UAV’s 3D trajectory, IoT devices’ transmit power, and scheduling. The main contributions of this paper are summarized as follows:

- First, we model a more accurate probabilistic LoS channel model to adapt the real-time changing characteristics of the channel. Under such a setup, we design the UAV’s 3D trajectory, IoT devices’ transmit power, and scheduling in the presence of a jammer. In addition, we consider the practical scenario, that is, the jammer with imperfect location information operates the process of transmitting jamming signals. This model fully considers the variability and complexity of the external environment, which has good practical value and research significance.
- Second, a UAV-enabled optimization framework is proposed under a probabilistic LoS channel, where a jammer with imperfect location information transmits jamming to the UAV. Specifically, we aim to maximize all IoT devices’ throughput based on the probabilistic LoS channel model. Because of the coupling of the variables, the non-convexity and non-concavity of the constraint, and the non-convexity of the objective function, it is intractable to solve the optimization problem. To this end, we first use the block coordinate descent (BCD) method to solve the coupling problem, which iteratively optimizes the UAV’s trajectory, IoT devices’ scheduling, and transmit power. Then, we apply the difference of convex (D.C) programming approach to address the non-convexity and non-concavity, and we address the integer UAV scheduling constraints by continuous relaxing. Because the sub-problems of optimizing the UAV trajectory are still non-convex, we utilize the successive convex approximation (SCA) technology and S-procedure to derive the local optimal solutions.
- Finally, we compare and verify different algorithms by simulation. Compared with the trajectory optimization based on the conventional simplified probabilistic LoS channel model, our proposed anti-jamming joint robust optimization algorithm based on the accurate probabilistic LoS model can vastly increase the system’s throughput by balancing the angle and distance trade-off. The performance is much better than the simplified probabilistic LoS channel model.

Next, we will introduce the system model, optimization problem, the proposed robust iterative algorithm and simulation comparison results in detail.

## 2. Problem Statement and Formulation

### 2.1. System Model

**Figure 1:**
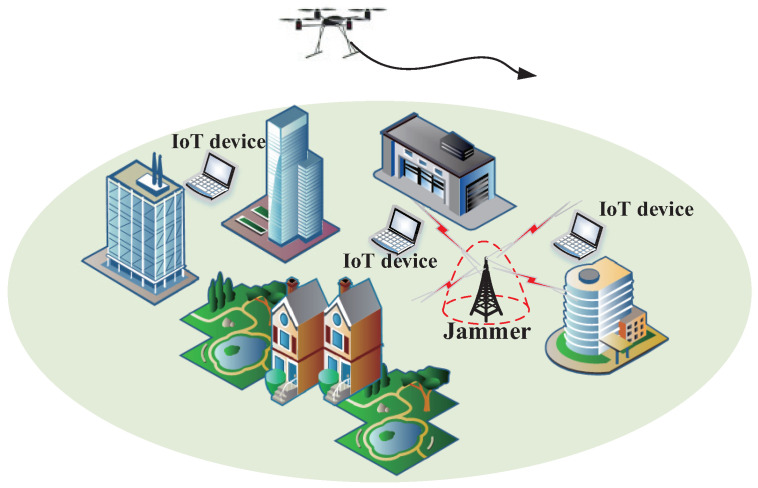
System model.

As shown in Figure 1, the UAV U provides services for K IoT devices. Given the complexity of the environment, there is a malicious jammer J sending a jamming signal with the intention of disrupting legitimate UAV communication. We express the set of IoT devices as $K=\left\{1,2,...K\right\}$, where $\left|K\right|=K$. Then the 3D position of the IoT devices K and J can be expressed as $q_{k}={\left[x_{k},y_{k},z_{k}\right]}^{†}\in R^{3\times 1}$, $q_{j}={\left[x_{j},y_{j},z_{j}\right]}^{†}\in R^{3\times 1}$, respectively. For a given task completion time *T*, we discretize the time into *N* equal time slots $n\in N\overset{\Delta }{\;=}\left\{1,...,N\right\}$, and each time slot Δt=TTNN is small enough. Therefore, the UAV trajectory can be expressed as the sequence of *N*, i.e., $Q\overset{\Delta }{\;=}\left\{q\left[n\right]={\left[x\left[n\right],y\left[n\right],z\left[n\right]\right]}^{†}\in R^{3\times 1},\forall n\right\}.$ We suppose that the UAV start from a given starting point $q^{start}$ and reach the ending point $q^{end}$ at the end of *T*, that is

(1) $$q\left[0\right]=q^{start},$$

(2) $$q\left[N\right]=q^{end},$$

Considering the UAV’s limited flight speed and flight altitude, the following constraints are given:

(3) $$∥q\left[n\right]-q\left[n-1\right]∥\leq V_{\max}\Delta t,$$

(4) $$H_{\min}\leq z\left[n\right]\leq H_{\max},\forall n,$$

where $V_{\max}$ is the UAV’s maximum flight speed; $H_{\min}$ and $H_{\max}$ are the UAV’s minimum and maximum flight altitudes, respectively.

The precise location of the jammer is unknown due to a lack of cooperation between the UAV and the malicious jammer. We assume the jammer’s uncertain region as a hemisphere [23], and the center of the hemisphere can be obtained as $q_{jc}=\left\{x_{jc},y_{jc},z_{jc}\right\}$, i.e., the jammer’s estimated position. Suppose that the errors between the estimated position and the exact position is $\left(\Delta x_{j},\Delta y_{j},\Delta z_{j}\right)\in \Delta q_{j}$. Hence, the jammer’s exact location is

(5) $$\begin{array}{c} x_{j}=x_{jc}+\Delta x_{j},\\ y_{j}=y_{jc}+\Delta y_{j},\\ z_{j}=z_{jc}+\Delta z_{j}, \end{array}$$

which is limited by

(6) $$\Delta q_{j}\overset{\Delta }{\;=}\left\{{\left(\Delta x_{j}\right)}^{2}+{\left(\Delta y_{j}\right)}^{2}+{\left(\Delta z_{j}\right)}^{2}\leq Q_{j}^{2},\Delta z_{j}\geq 0\right\},$$

where $Q_{j}$ is the radius of the hemisphere.

Assume that each IoT device can only transmit data to the UAV when it is scheduled. Define a binary variable $A\overset{\Delta }{\;=}\left\{a_{k}\left[n\right],\forall k,n\right\}$, which indicates whether IoT device *k* plans to transmit information to the UAV in time slot *n*. If the IoT device *k* sends information, that is $a_{k}\left[n\right]=1$, otherwise $a_{k}\left[n\right]=0$. Assuming that only one IoT device is allowed to transmit information to the UAV in each time slot, the scheduling constraints are as follows:

(7) $$\sum_{k=1}^{K}a_{k}\left[n\right]\leq 1,\forall n,$$

(8) $$a_{k}\left[n\right]\in \left\{0,1\right\},\forall k,n.$$

We assume that the transmit power of the IoT devices in the *n*th time slot is $P\overset{\Delta }{\;=}\left\{p_{k}\left[n\right],\forall k,n\right\}$, and due to the IoT devices’ power limitation, it satisfies the following constraints:

(9) $$\frac{1}{N}\sum_{n=1}^{N}p_{k}\left[n\right]\leq \bar{p},\forall k,$$

(10) $$p_{k}\left[n\right]\leq p_{max},\forall k,n,$$

where $\bar{p}$ is the IoT devices’ average transmit power and $p_{max}$ is the IoT devices’ maximum transmit power. Constraint (9) denotes that the IoT device has a limited total energy $N\bar{p}$.

### 2.2. Channel Model

We model the U2G communication link as a probabilistic LoS (PL) channel. In time slot *n*, the LoS probability of the PL channel can be modeled as

(11) $$P_{i}^{L}\left[n\right]=\frac{1}{1+be^{\left(-c\left[\theta_{i}\left[n\right]-b\right]\right)}},i\in \left\{k,j\right\},\forall n,$$

where $i\in \left\{k,j\right\}$, b>0, and c>0 are constants determined by the practical environment. The NLoS probability of the PL channel can be modeled as

(12) $$P_{i}^{N}\left[n\right]=1-P_{i}^{L}\left[n\right].$$

The elevation angle from device *k* to UAV in time slot *n* can be calculated as

(13) $$\theta_{i}\left[n\right]=\frac{180}{\pi }\arcsin\left(\frac{z\left[n\right]}{d_{i}\left[n\right]}\right),i\in \left\{k,j\right\},\forall n,$$

where $d_{i}\left[n\right]=\sqrt{{∥q\left[n\right]-q_{i}\left[n\right]∥}^{2}}$ is the distance between the UAV and the device *i*. Then, the channel gain between UAV and device *i* can be expressed as

(14) $$g_{i}^{L}\left[n\right]=\beta_{0}d_{i}^{-\alpha_{L}}\left[n\right],$$

(15) $$g_{i}^{N}\left[n\right]=\mu \beta_{0}d_{i}^{-\alpha_{N}}\left[n\right],\mu <1,$$

where $\beta_{0}$ represents the average channel power gain at the reference distance $d_{0}=1$, μ<1 is the additional signal attenuation factor under the NLoS link; $\alpha^{L}$ and $\alpha^{N}$ represent the average path loss exponents for the LoS channel and NLoS channel, respectively.

### 2.3. Problem Formulation for Throughput Maximization

We assume that the U2G channel is allocated with a unit bandwidth [36]. According to the PL channel model, the uplink throughput from the scheduled IoT device *k* to the UAV in time slot *n* can be given by

(16) $$R_{k}\left[n\right]=\log_{2}\left(1+\frac{g_{k}^{I}\left[n\right]p_{k}\left[n\right]}{\sigma^{2}+g_{j}^{I}\left[n\right]p_{j}}\right),I\in \left\{L,N\right\},\forall k$$

where $I\in \left\{L,N\right\}$, $\sigma^{2}$ denotes the power of additive white Gaussian noise (AWGN) at the UAV, and $p_{j}$ denotes the jamming power.

In a statistical sense, the expected instantaneous throughput under the PL channel model from IoT device *k* to the UAV in time slot *n* can be given by

(17) $$\begin{array}{c} E\left[R_{k}\left[n\right]\right]=P_{k}^{L}\left[n\right]P_{j}^{L}\left[n\right]R_{k}^{LL}\left[n\right]+P_{k}^{L}\left[n\right]P_{j}^{N}\left[n\right]R_{k}^{LN}\left[n\right]\\ \;\;\;\;\;\;+P_{k}^{N}\left[n\right]P_{j}^{L}\left[n\right]R_{k}^{NL}\left[n\right]+P_{k}^{N}\left[n\right]P_{j}^{N}\left[n\right]R_{k}^{NN}\left[n\right],\forall k, \end{array}$$

where

$$R_{k}^{LL}\left[n\right]=\log_{2}\left(1+\frac{\gamma_{0}d_{k}^{-\alpha_{L}}\left[n\right]p_{k}\left[n\right]}{1+\gamma_{0}d_{k}^{-\alpha_{L}}\left[n\right]p_{j}}\right),\forall k,$$

$$R_{k}^{LN}\left[n\right]=\log_{2}\left(1+\frac{\gamma_{0}d_{k}^{-\alpha_{L}}\left[n\right]p_{k}\left[n\right]}{1+\gamma_{0}\mu d_{k}^{-\alpha_{N}}\left[n\right]p_{j}}\right),\forall k,$$

$$R_{k}^{NL}\left[n\right]=\log_{2}\left(1+\frac{\gamma_{0}\mu d_{k}^{-\alpha_{N}}\left[n\right]p_{k}\left[n\right]}{1+\gamma_{0}d_{k}^{-\alpha_{L}}\left[n\right]p_{j}}\right),\forall k,$$

$$R_{k}^{NN}\left[n\right]=\log_{2}\left(1+\frac{\gamma_{0}\mu d_{k}^{-\alpha_{N}}\left[n\right]p_{k}\left[n\right]}{1+\gamma_{0}\mu d_{k}^{-\alpha_{N}}\left[n\right]p_{j}}\right),\forall k,$$

and

$$\gamma_{0}=\frac{\beta_{0}}{\sigma^{2}}.$$

Equation (16) denotes the achievable rates at the UAV conditioned on the LoS and NLoS states of the U2G channel. Thus, the expected instantaneous throughput in (17) can be approximated by its lower bound as

(18) $$\begin{array}{c} E\left[R_{k}\left[n\right]\right]\geq P_{k}^{L}\left[n\right]P_{j}^{L}\left[n\right]R_{k}^{LL}\left[n\right]+P_{k}^{L}\left[n\right]P_{j}^{N}\left[n\right]R_{k}^{LN}\left[n\right]\\ \;\;\;\;\;\;\;\;\;\;\;\;\;\;+P_{k}^{N}\left[n\right]P_{j}^{N}\left[n\right]R_{k}^{NN}\left[n\right]\overset{\Delta }{\;=}{\bar{R}}_{k}\left[n\right]. \end{array}$$

**Lemma** **1.** *From (14) and (15), we can observe that, given the UAV’s location, the instantaneous throughput in the NLoS state is practically much smaller than that in the LoS state due to the additional signal attenuation μ and a larger path loss exponent $\alpha_{N}$. To achieve a more accurate approximation, we can use (18) instead of (17). Their equivalence can be proved in [34], specifically, setting the system parameters as μ=20 dB, $\alpha_{L}=2.5$, $\alpha_{N}=3.5$, $\beta_{0}=-60$ dB, the achievable rates in the LoS and NLoS states are 5.85 bps/Hz and 0.016 bps/Hz, respectively. Hence, we can draw the following comparison:*

(19) $$R_{k}^{LN}>R_{k}^{LL}\approx R_{k}^{NN}\gg R_{k}^{NL}$$

Therefore, to reduce complexity, we use ${\bar{R}}_{k}\left[n\right]$ instead of $E\left[R_{k}\left[n\right]\right]$.

Our goal is to maximize the data throughput of all IoT devices in *T*. Based on (18), the corresponding optimization problem can be expressed as

(20a) $$\begin{array}{cc} & \;\;\;\;\;\;\;\max_{\left\{Q,A,P,\theta_{i}\right\}}\sum_{n=1}^{N}\sum_{k=1}^{K}a_{k}\left[n\right]{\bar{R}}_{k}\left[n\right]\; \end{array}$$

(20b) $$\begin{array}{cc} & \;\;\;\;\;\;\;\;\;\;\;\;\;\;\;\;\;\;s.t.q\left[0\right]=q^{start},\; \end{array}$$

(20c) $$\begin{array}{cc} & \;\;\;\;\;\;\;\;\;\;\;\;\;\;\;\;\;\;\;\;\;\;\;\;\;q\left[N\right]=q^{end},\; \end{array}$$

(20d) $$\begin{array}{cc} & \;\;\;\;\;\;\;\;\;\;\;\;\;\;\;\;\;\;\;\;\;\;\;\;\;∥q\left[n\right]-q\left[n-1\right]∥\leq V_{\max}\Delta t,\; \end{array}$$

(20e) $$\begin{array}{cc} & \;\;\;\;\;\;\;\;\;\;\;\;\;\;\;\;\;\;\;\;\;\;\;\;\;H_{\min}\leq z\left[n\right]\leq H_{\max},\forall n,\; \end{array}$$

(20f) $$\begin{array}{cc} & \;\;\;\;\;\;\;\;\;\;\;\;\;\;\;\;\;\;\;\;\;\;\;\;\;\frac{1}{N}\sum_{n=1}^{N}p_{k}\left[n\right]\leq \bar{p},\forall k,\; \end{array}$$

(20g) $$\begin{array}{cc} & \;\;\;\;\;\;\;\;\;\;\;\;\;\;\;\;\;\;\;\;\;\;\;\;\;p_{k}\left[n\right]\leq p_{max},\forall k,n,\; \end{array}$$

(20h) $$\begin{array}{cc} & \;\;\;\;\;\;\;\;\;\;\;\;\;\;\;\;\;\;\;\;\;\;\;\;\;\sum_{k=1}^{K}a_{k}\left[n\right]\leq 1,\forall n,\; \end{array}$$

(20i) $$\begin{array}{cc} & \;\;\;\;\;\;\;\;\;\;\;\;\;\;\;\;\;\;\;\;\;\;\;\;\;a_{k}\left[n\right]\in \left\{0,1\right\},\forall k,n.\; \end{array}$$

(20j) $$\begin{array}{cc} & \;\;\;\;\;\;\;\;\;\;\;\;\;\;\;\;\;\;\;\;\;\;\;\;\;\theta_{i}\left[n\right]=\frac{180}{\pi }\arcsin\left(\frac{z\left[n\right]}{d_{i}\left[n\right]}\right),i\in \left\{k,j\right\},\forall n,\; \end{array}$$

In the objective function of (20), ${\bar{R}}_{k}\left[n\right]$ is non-convex with respect to Q. Therefore, we introduce a slack variable η, and (20) can be reconstructed as follows:

(21a) $$\begin{array}{cc} & \;\;\;\;\max_{\left\{Q,A,P,\theta_{i},\eta \right\}}\eta \; \end{array}$$

(21b) $$\begin{array}{cc} & \;\;\;\;\;\;\;\;\;\;\;\;\;\;\;\;s.t.\sum_{n=1}^{N}\sum_{k=1}^{K}a_{k}\left[n\right]{\bar{R}}_{k}\left[n\right]\geq \eta ,\; \end{array}$$

    (20b)−(20j).

In (21), the function in (21b) is non-convex, and the optimization variables Q, A, and P are coupled. The variables for IoT devices’ scheduling in (20h) and (20i) are binary, and (20j) is a non-affine constraint. Therefore, (21) is a mixed integer nonlinear problem, there is usually no effective optimal method to get its optimal solution, and it is intractable to work it out.

## 3. Robust Iterative Algorithm for Throughput Maximization

To proceed, we transform the original optimization problem (21) into a more efficient form. First, by using the BCD method, we decouple the trajectory Q, scheduling A, and transmit power P into three blocks. In addition, we use the SCA technique to solve the non-convex problem. To be specific, the UAV trajectory, IoT devices’ transmit power, and scheduling variables are iteratively optimized in each iteration until the algorithm converges.

### 3.1. Trajectory Optimization with Given Scheduling and Transmit Power

Given any feasible IoT devices’ scheduling A and transmit power P, (21) reduces to the following UAV trajectory optimization problem:

(22a) $$\begin{array}{cc} & \;\;\;\;\;\;\;\;\;\;\;\;\;\;\max_{\left\{Q,\theta_{i},\eta \right\}}\eta \; \end{array}$$

(22b) $$\begin{array}{cc} & \;\;\;\;\;\;\;\;\;\;\;\;\;\;\;\;\;\;\;\;s.t.\sum_{n=1}^{N}\sum_{k=1}^{K}a_{k}\left[n\right]{\bar{R}}_{k}\left[n\right]\geq \eta ,\; \end{array}$$

    (20b)−(20e),(20j).

To solve the non-convexity of (22b), we introduce auxiliary variables $B_{i}$, $C_{i}$, and $D_{i}$, i.e.,

(23) $$B_{i}=\left\{B_{i}\left[n\right],\forall i\in \left\{k,j\right\}\right\},$$

(24) $$C_{i}=\left\{C_{i}\left[n\right],\forall i\in \left\{k,j\right\}\right\},$$

(25) $$D_{i,I}=\left\{D_{i,I}\left[n\right],\forall i\in \left\{k,j\right\},I\in \left\{L,N\right\}\right\},$$

Then, ${\bar{R}}_{k}\left[n\right]$ is transformed into the following equivalent form:

(26) $$\begin{array}{c} {\bar{R}}_{k}\left[n\right]=\frac{1}{B_{k}\left[n\right]B_{j}\left[n\right]}\log_{2}\left(1+\frac{\gamma_{0}p_{k}\left[n\right]D_{k,L}^{-1}\left[n\right]}{1+\gamma_{0}p_{j}D_{j,L}^{-1}\left[n\right]}\right)+\\ \;\;\;\;\;\;\;\;\;\;\;\frac{1}{B_{k}\left[n\right]C_{j}\left[n\right]}\log_{2}\left(1+\frac{\gamma_{0}p_{k}\left[n\right]D_{k,L}^{-1}\left[n\right]}{1+\gamma_{0}\mu p_{j}D_{j,N}^{-1}\left[n\right]}\right)+\\ \;\;\;\;\;\;\;\;\;\;\;\frac{1}{C_{k}\left[n\right]C_{j}\left[n\right]}\log_{2}\left(1+\frac{\gamma_{0}\mu p_{k}\left[n\right]D_{k,N}^{-1}\left[n\right]}{1+\gamma_{0}\mu p_{j}D_{j,N}^{-1}\left[n\right]}\right),\forall k, \end{array}$$

where

(27) $$B_{i}\left[n\right]\geq 1+be^{\left(-c\left[\theta_{i}\left[n\right]-b\right]\right)},$$

(28) $$C_{i}\left[n\right]\geq 1+\frac{1}{b}e^{\left(c\left[\phi_{i}\left[n\right]-b\right]\right)},$$

(29) $$D_{k,I}\left[n\right]\geq {∥q\left[n\right]-q_{k}∥}^{\alpha_{I}},$$

(30) $$D_{j,I}\left[n\right]\leq \min_{\Delta q}{∥q\left[n\right]-q_{j}∥}^{\alpha_{I}}.$$

and

(31) $$\theta_{i}\left[n\right]\leq \frac{180}{\pi }\arcsin\left(\frac{z\left[n\right]}{d_{i}\left[n\right]}\right),i\in \left\{k,j\right\},\forall n,$$

(32) $$\phi_{i}\left[n\right]\geq \frac{180}{\pi }\arcsin\left(\frac{z\left[n\right]}{d_{i}\left[n\right]}\right),i\in \left\{k,j\right\},\forall n.$$

For constraint (30), the uncertainty of the jammer’s position leads to an infinite number of variables Δq. Leveraging (5) and (6), constraint (30) is equivalent to

(33a) $$\begin{array}{cc} & \;\;\;\;\;\;\;\;\;\;\;\;\;\;\;\;\;\;\;\;\;\;\;\;\;\;\;\;\;\;\;\;\;\;\;\;\;\;\;\;\;\;\;\;\;\;\;\;\;\;\;\;\;\;\;\;\;\;\;\;\;{\left(\Delta x_{j}\right)}^{2}+{\left(\Delta y_{j}\right)}^{2}+{\left(\Delta z_{j}\right)}^{2}\leq Q_{j}^{2},\; \end{array}$$

(33b) $$\begin{array}{cc} & \;\;\;\;\;\;\;\;\;\;\;\;\;\;\;\;\;\;\;\;\;\;\;\;\;\;\;\;\;\;\;\;\;\;\;\;\;\;\;\;\;\;\;\;\;\;\;\;\;\;\begin{array}{c} D_{j,I}\left[n\right]\leq {\left(x_{jc}+\Delta x_{j}-x\left[n\right]\right)}^{2}+{\left(y_{jc}+\Delta y_{j}-y\left[n\right]\right)}^{2}\\ \;\;\;\;\;\;\;\;\;\;\;\;+{\left(z_{jc}+\Delta z_{j}-z\left[n\right]\right)}^{2},\forall n. \end{array}\; \end{array}$$

*S-Procedure* [37]: Denote F,r, and *h* as the m×m symmetric matrix, *m* dimensional column vector, and real number, respectively. Suppose there is $\hat{\vartheta }$ with

(34) $${\hat{\vartheta }}^{†}F_{1}\hat{\vartheta }+2r_{1}^{†}\hat{\vartheta }+h_{1}<0.$$

Then, there is ϑ with

(35) $$\begin{array}{c} \vartheta^{†}F_{1}\vartheta +2r_{1}^{†}\vartheta +h_{1}\leq 0,\\ \vartheta^{†}F_{2}\vartheta +2r_{2}^{†}\vartheta +h_{2}\leq 0, \end{array}$$

if and only if there is a $\lambda \underline{≻}0$ such that

(36) $$\lambda \left[\begin{array}{cc} F_{1} & r_{1}\\ r_{1}^{†} & h_{1} \end{array}\right]\underline{≻}\left[\begin{array}{cc} F_{2} & r_{2}\\ r_{2}^{†} & h_{2} \end{array}\right].$$

Thus, we can hold the following implication:

(37) $$\vartheta^{†}F_{1}\vartheta +2r_{1}^{†}\vartheta +h_{1}\leq 0\Rightarrow \vartheta^{†}F_{2}\vartheta +2r_{2}^{†}\vartheta +h_{2}\leq 0.$$

It can be observed that ${\left(\Delta x_{j}\right)}^{2}+{\left(\Delta y_{j}\right)}^{2}+{\left(\Delta z_{j}\right)}^{2}-Q_{j}^{2}\leq 0,$ with $\left(\Delta x_{j},\Delta y_{j},\Delta z_{j}\right)=\left(0,0,0\right)$. Taking advantage of the S-Procedure described above, (33a) implies (33b) if and only if $\Gamma \left(x\left[n\right],y\left[n\right],z\left[n\right],D_{j,I}\left[n\right],\delta_{j}\left[n\right]\right)\underline{≻}0,\forall n,$ where $\delta_{j}\left[n\right]\geq 0,$ and

(38) $$\begin{array}{c} \Gamma (x\left[n\right],y\left[n\right],z\left[n\right],D_{j,I}\left[n\right],\delta_{j}\left[n\right])=\\ \left[\begin{array}{cccc} \delta_{j}\left[n\right]+1 & 0 & 0 & x_{jc}-x\left[n\right]\\ 0 & \delta_{j}\left[n\right]+1 & 0 & y_{jc}-y\left[n\right]\\ 0 & 0 & \delta_{j}\left[n\right]+1 & z_{jc}-z\left[n\right]\\ x_{jc}-x\left[n\right] & y_{jc}-y\left[n\right] & z_{jc}-z\left[n\right] & \varphi_{j}\left[n\right] \end{array}\right],\forall n, \end{array}$$

with

(39) $$\begin{array}{c} \varphi_{j}\left[n\right]=-\delta_{j}\left[n\right]Q_{j}^{2}+{\left(x_{jc}-x\left[n\right]\right)}^{2}+{\left(y_{jc}-y\left[n\right]\right)}^{2}\\ \;\;\;\;\;\;\;\;\;\;+{\left(z_{jc}-z\left[n\right]\right)}^{2}-D_{j,I}\left[n\right],\forall n. \end{array}$$

However, (38) is non-convex, and it is troublesome to solve it. To proceed, we utilize the first-order Taylor expansion to obtain the lower bound of Equation (38) at a given point. At the given point $(x{\left[n\right]}^{f},y{\left[n\right]}^{f},z{\left[n\right]}^{f}),$ (39) can be reformulated as

(40) $$\begin{array}{c} \varphi_{j}\left[n\right]\geq {\bar{\varphi }}_{j}\left[n\right]=\\ -\delta_{j}\left[n\right]Q_{j}^{2}+x_{jc}^{2}-2x_{jc}x\left[n\right]+x^{2}{\left[n\right]}^{f}\\ +2x{\left[n\right]}^{f}\left(x\left[n\right]-x{\left[n\right]}^{f}\right)+y_{jc}^{2}-2y_{jc}y\left[n\right]+y^{2}{\left[n\right]}^{f}\\ +2y{\left[n\right]}^{f}\left(y\left[n\right]-y{\left[n\right]}^{f}\right)+z_{jc}^{2}-2z_{jc}z\left[n\right]+z^{2}{\left[n\right]}^{f}\\ +2z{\left[n\right]}^{f}\left(z\left[n\right]-z{\left[n\right]}^{f}\right)-D_{j,I}\left[n\right],\forall n. \end{array}$$

Then, according to the Lemma 1 in [26], it can be known that the term

(41) $$\tilde{\Gamma }\left(x\left[n\right],y\left[n\right],z\left[n\right],D_{j,I}\left[n\right],\delta_{j}\left[n\right]\right)\underline{≻}0$$

implies that

(42) $$\Gamma \left(x\left[n\right],y\left[n\right],z\left[n\right],D_{j,I}\left[n\right],\delta_{j}\left[n\right]\right)\underline{≻}0,$$

where

(43) $$\begin{array}{c} \tilde{\Gamma }\left(x\left[n\right],y\left[n\right],z\left[n\right],D_{j,I}\left[n\right],\delta_{j}\left[n\right]\right)=\\ \left[\begin{array}{cccc} \delta_{j}\left[n\right]+1 & 0 & 0 & x_{jc}-x\left[n\right]\\ 0 & \delta_{j}\left[n\right]+1 & 0 & y_{jc}-y\left[n\right]\\ 0 & 0 & \delta_{j}\left[n\right]+1 & z_{jc}-z\left[n\right]\\ x_{jc}-x\left[n\right] & y_{jc}-y\left[n\right] & z_{jc}-z\left[n\right] & {\bar{\varphi }}_{j}\left[n\right] \end{array}\right]. \end{array}$$

To handle the non-affine constraint (20j), we have its relaxed constraints as (31) and (32). It can be proved that (26)–(30), (31), and (32) must maintain the equal sign [38], and there must be $\theta_{i}\left[n\right]=\phi_{i}\left[n\right]$ to ensure that the value at (22) does not decrease.

Now, (22b) is still not convex or concave, for which the optimal solution is challenging to obtain. Note that the objective is the difference of two functions; using the D.C programming approach, the non-convex and non-concave function ${\bar{R}}_{k}$ can be transformed into the following equivalent form:

$${\bar{R}}_{k}={\bar{R}}_{k}^{+}\left[n\right]-{\bar{R}}_{k}^{-}\left[n\right],$$

where

(44) $$\begin{array}{c} {\bar{R}}_{k}^{+}\left[n\right]\overset{\Delta }{\;=}f_{1}+f_{2}+f_{3}\\ \;\;\;\;\;\;\;\;=\frac{1}{B_{k}\left[n\right]B_{j}\left[n\right]}\log_{2}\left(1+\frac{\gamma_{0}p_{k}\left[n\right]}{D_{k,L}\left[n\right]}+\frac{\gamma_{0}p_{j}}{D_{j,L}\left[n\right]}\right)+\\ \;\;\;\;\;\;\;\;\;\;\;\;\frac{1}{B_{k}\left[n\right]C_{j}\left[n\right]}\log_{2}\left(1+\frac{\gamma_{0}p_{k}\left[n\right]}{D_{k,L}\left[n\right]}+\frac{\gamma_{0}\mu p_{j}}{D_{j,N}\left[n\right]}\right)+\\ \;\;\;\;\;\;\;\;\;\;\;\;\frac{1}{C_{k}\left[n\right]C_{j}\left[n\right]}\log_{2}\left(1+\frac{\gamma_{0}\mu p_{k}\left[n\right]}{D_{k,N}\left[n\right]}+\frac{\gamma_{0}\mu p_{j}}{D_{j,N}\left[n\right]}\right), \end{array}$$

(45) $$\begin{array}{c} {\bar{R}}_{k}^{-}\left[n\right]=\frac{1}{B_{k}\left[n\right]B_{j}\left[n\right]}\log_{2}\left(1+\frac{\gamma_{0}p_{j}}{D_{j,L}\left[n\right]}\right)+\\ \;\;\;\;\;\;\;\;\;\;\;\;\frac{1}{B_{k}\left[n\right]C_{j}\left[n\right]}\log_{2}\left(1+\frac{\gamma_{0}\mu p_{j}}{D_{j,N}\left[n\right]}\right)+\\ \;\;\;\;\;\;\;\;\;\;\;\;\frac{1}{C_{k}\left[n\right]C_{j}\left[n\right]}\log_{2}\left(1+\frac{\gamma_{0}\mu p_{j}}{D_{j,N}\left[n\right]}\right). \end{array}$$

**Lemma** **2.** *Let*

(46) $$f\left(\xi_{1},\xi_{2},\xi_{3},\xi_{4}\right)=\frac{1}{\xi_{1}}\frac{1}{\xi_{2}}\log_{2}\left(1+\frac{\Lambda }{\xi_{3}}+\frac{\Xi }{\xi_{4}}\right).$$

*Given Λ>0 and Ξ>0, function $f\left(\xi_{1},\xi_{2},\xi_{3},\xi_{4}\right)$ is jointly convex with respect to (w.r.t.) its positive variables. Thus, by applying the first-order Taylor expansion at any given point $f^{f}$, its lower bounds can be approximated as*

(47) $$\begin{array}{c} f\geq f^{f}+f_{\xi_{1}}^{f}\left(\xi_{1}-\xi_{1}^{f}\right)+f_{\xi_{2}}^{f}\left(\xi_{2}-\xi_{2}^{f}\right)\\ +f_{\xi_{3}}^{f}\left(\xi_{3}-\xi_{3}^{f}\right)+f_{\xi_{4}}^{f}\left(\xi_{4}-\xi_{4}^{f}\right) \end{array}$$

*where*

$$f^{f}=\frac{1}{\xi_{1}^{f}}\frac{1}{\xi_{2}^{f}}\log_{2}\left(1+\frac{\Lambda }{\xi_{3}^{f}}+\frac{\Xi }{\xi_{4}^{f}}\right),$$

$$f_{\xi_{1}}^{f}=-\frac{\log_{2}\left(1+\frac{\Lambda }{\xi_{3}^{f}}+\frac{\Xi }{\xi_{4}^{f}}\right)}{{\left(\xi_{1}^{f}\right)}^{2}\xi_{2}^{f}},$$

$$f_{\xi_{2}}^{f}=-\frac{\log_{2}\left(1+\frac{\Lambda }{\xi_{3}^{f}}+\frac{\Xi }{\xi_{4}^{f}}\right)}{\xi_{1}^{f}{\left(\xi_{2}^{f}\right)}^{2}},$$

$$f_{\xi_{3}}^{f}=-\frac{\Lambda \log_{2}e}{\xi_{1}^{f}\xi_{2}^{f}{\left(\xi_{3}^{f}\right)}^{2}\left(1+\frac{\Lambda }{\xi_{3}^{f}}+\frac{\Xi }{\xi_{4}^{f}}\right)},$$

$$f_{\xi_{4}}^{f}=-\frac{\Xi \log_{2}e}{\xi_{1}^{f}\xi_{2}^{f}{\left(\xi_{4}^{f}\right)}^{2}\left(1+\frac{\Lambda }{\xi_{3}^{f}}+\frac{\Xi }{\xi_{4}^{f}}\right)}.$$

**Proof.**  Please see Appendix A in [35].    □

For ${\bar{R}}_{k}^{+}\left[n\right]$, all terms are convex w.r.t. their corresponding variables. Thus, on the basis of Lemma 2, ${\bar{R}}_{k}^{+}\left[n\right]$ can be approximated by its global lower bound as

(48) $$\begin{array}{c} {\bar{R}}_{k}^{+}\left[n\right]\geq {\bar{R}}_{k}^{+,lb}\left[n\right]\overset{\Delta }{\;=}f_{1}^{f}+f_{2}^{f}+f_{3}^{f}\\ \;\;\;\;\;\;\;\;\;\;\;+\left(B_{k}\left[n\right]-B_{k}^{f}\left[n\right]\right)\left(f_{1,B_{k}}^{f}+f_{2,B_{k}}^{f}\right)\\ \;\;\;\;\;\;\;\;\;\;\;+\left(B_{j}\left[n\right]-B_{j}^{f}\left[n\right]\right)f_{1,B_{j}}^{f}\\ \;\;\;\;\;\;\;\;\;\;\;+\left(C_{j}\left[n\right]-C_{j}^{f}\left[n\right]\right)\left(f_{2,C_{j}}^{f}+f_{3,C_{j}}^{f}\right)\\ \;\;\;\;\;\;\;\;\;\;\;+\left(C_{k}\left[n\right]-C_{k}^{f}\left[n\right]\right)f_{3,C_{k}}^{f}\\ \;\;\;\;\;\;\;\;\;\;\;+\left(D_{j,L}\left[n\right]-D_{j,L}^{f}\left[n\right]\right)f_{1,D_{j,L}}^{f}\\ \;\;\;\;\;\;\;\;\;\;\;+\left(D_{k,L}\left[n\right]-D_{k,L}^{f}\left[n\right]\right)\left(f_{1,D_{k,L}}^{f}+f_{2,D_{k,L}}^{f}\right)\\ \;\;\;\;\;\;\;\;\;\;\;+\left(D_{j,N}\left[n\right]-D_{j,N}^{f}\left[n\right]\right)\left(f_{2,D_{j,N}}^{f}+f_{3,D_{j,N}}^{f}\right)\\ \;\;\;\;\;\;\;\;\;\;\;+\left(D_{k,N}\left[n\right]-D_{k,N}^{f}\left[n\right]\right)f_{3,D_{k,N}}^{f},\forall k,n. \end{array}$$

Next, we will further handle the complicated terms in ${\bar{R}}_{k}^{-}\left[n\right]$. Define

(49) $$\Omega =\left\{\Omega_{1,k}\left[n\right],\Omega_{2,k}\left[n\right],\Omega_{3,k}\left[n\right],\forall k,n\right\},$$

then ${\bar{R}}_{k}$ can be equivalently expressed as

(50) $${\bar{R}}_{k}\left[n\right]\geq {\bar{R}}_{k}^{+,lb}\left[n\right]-\Omega_{1,k}\left[n\right]-\Omega_{2,k}\left[n\right]-\Omega_{3,k}\left[n\right],$$

where

(51a) $$\begin{array}{c} \Omega_{1,k}\left[n\right]\geq \frac{1}{B_{k}\left[n\right]B_{j}\left[n\right]}\omega_{1}\left[n\right], \end{array}$$

(51b) $$\begin{array}{c} \Omega_{2,k}\left[n\right]\geq \frac{1}{B_{k}\left[n\right]C_{j}\left[n\right]}\omega_{2}\left[n\right], \end{array}$$

(51c) $$\begin{array}{c} \Omega_{3,k}\left[n\right]\geq \frac{1}{C_{k}\left[n\right]C_{j}\left[n\right]}\omega_{2}\left[n\right]. \end{array}$$

For (51), the equation must be maintained, otherwise ${\bar{R}}_{k}\left[n\right]$ will be reduced. Then, we solve it by applying the first-order Taylor expansion at any feasible point. Letting $\omega^{f}=\left\{\omega_{1}^{f}\left[n\right],\omega_{2}^{f}\left[n\right],\forall n\right\}$, we have the following equations:

(52a) $$\begin{array}{c} \begin{array}{c} \log_{2}\left(B_{k}\left[n\right]\right)+\log_{2}\left(B_{j}\left[n\right]\right)+\log_{2}\left(\Omega_{1,k}\left[n\right]\right)\geq \\ \log_{2}\left(\omega_{1}^{f}\left[n\right]\right)+\frac{\omega_{1}\left[n\right]-\omega_{1}^{f}\left[n\right]}{\omega_{1}^{f}\left[n\right]\mathrm{In}2}, \end{array} \end{array}$$

(52b) $$\begin{array}{c} \begin{array}{c} \log_{2}\left(B_{k}\left[n\right]\right)+\log_{2}\left(C_{j}\left[n\right]\right)+\log_{2}\left(\Omega_{2,k}\left[n\right]\right)\geq \\ \log_{2}\left(\omega_{2}^{f}\left[n\right]\right)+\frac{\omega_{2}\left[n\right]-\omega_{2}^{f}\left[n\right]}{\omega_{2}^{f}\left[n\right]\mathrm{In}2}, \end{array} \end{array}$$

(52c) $$\begin{array}{c} \begin{array}{c} \log_{2}\left(C_{k}\left[n\right]\right)+\log_{2}\left(C_{j}\left[n\right]\right)+\log_{2}\left(\Omega_{3,k}\left[n\right]\right)\geq \\ \log_{2}\left(\omega_{2}^{f}\left[n\right]\right)+\frac{\omega_{2}\left[n\right]-\omega_{2}^{f}\left[n\right]}{\omega_{2}^{f}\left[n\right]\mathrm{In}2}, \end{array} \end{array}$$

where

$$\omega_{1}\geq \log_{2}\left(1+e^{v_{1}\left[n\right]}\right),$$

$$\omega_{2}\geq \log_{2}\left(1+e^{v_{2}\left[n\right]}\right).$$

(53a) $$\begin{array}{c} D_{j,L}\left[n\right]\geq \gamma_{0}p_{j}e^{-v_{1}\left[n\right]}, \end{array}$$

(53b) $$\begin{array}{c} D_{j,N}\left[n\right]\geq \gamma_{0}\mu p_{j}e^{-v_{2}\left[n\right]}. \end{array}$$

Then, we can observe that (30) and (31) are non-convex. For (30), using first-order Taylor expansion, we have

(54) $$\begin{array}{c} D_{j,I}\left[n\right]\leq {∥q^{f}\left[n\right]-q_{j}∥}^{\alpha_{I}}+\alpha_{I}{∥q^{f}\left[n\right]-q_{j}∥}^{\alpha_{I}-2}\\ \;\;\;\;\;\;\;\;\;\;\;\;\;{\left(q^{f}\left[n\right]-q_{j}\right)}^{†}\left(q\left[n\right]-q^{f}\left[n\right]\right). \end{array}$$

For (31), it is non-convex, and is convex w.r.t. $d_{i}\left[n\right]$. Therefore, (31) can be transformed into

**Lemma** **3.** *At any local point $\zeta^{f}$, we can obtain the following constraint with the aid of the first-order Taylor expansion:*

(55) $$\begin{array}{c} \theta_{i}\left[n\right]\leq \frac{180}{\pi }\arcsin\left(\frac{z\left[n\right]}{∥q\left[n\right]-q_{i}\left[n\right]∥}\right)\\ \;\;\;\;\;\;=-\frac{180}{\pi }G_{i}^{f}\left[n\right]\left(∥q\left[n\right]-q_{i}\left[n\right]∥-∥q^{f}\left[n\right]-q_{i}\left[n\right]∥\right)\\ \;\;\;\;\;\;\;\;\;\;\;+\frac{180}{\pi }F_{i}^{f}\left[n\right],i\in \left\{k,j\right\},\forall n, \end{array}$$

*where*

$$F_{i}^{f}\left[n\right]=\arcsin\left(\frac{z^{f}\left[n\right]}{∥q^{f}\left[n\right]-q_{i}\left[n\right]∥}\right),$$

$$G_{i}^{f}\left[n\right]=\frac{z^{f}\left[n\right]}{{∥q^{f}\left[n\right]-q_{i}\left[n\right]∥}^{2}}.$$

**Proof.**  Please see Lemma 3 in [34].    □

Now, the optimization (22) can be reformulated into the following convex problem:

(56a) $$\begin{array}{cc} & \;\max_{\left\{Q,\theta_{i},\phi_{i},B_{i},C_{i},D_{i,I},\omega ,\Omega ,v,\eta \right\}}\eta \; \end{array}$$

(56b) $$\begin{array}{cc} & \;\;\;\;\;\;\;s.t.\sum_{n=1}^{N}\sum_{k=1}^{K}a_{k}\left[n\right]\left({\bar{R}}_{k}^{+,lb}\left[n\right]-\Omega_{1}\left[n\right]-\Omega_{2}\left[n\right]-\Omega_{3}\left[n\right]\right)\geq \eta ,\; \end{array}$$

    (20b)−(20e),(27)−(29),(32),(52)−(55).

Problem (56) is a semidefinite optimization problem. We can solve it by CVX [39]. In addition, as (52), (54), and (55) used the SCA technology, (56) provides a lower bound of the original optimization problem (22).

### 3.2. Scheduling Optimization with Given Transmit Power and Trajectory

For given trajectory Q and transmit power P, we first relax the binary variable A into continuous variable, i.e.,

(57) $$\tilde{A}\overset{\Delta }{\;=}\left\{0\leq {\tilde{a}}_{k}\left[n\right]\leq 1,\forall k,n\right\},$$

Then, optimization problem (21) can be transformed into the following form:

(58a) $$\begin{array}{cc} & \;\;\;\;\;\;\;\max_{\left\{A,\eta \right\}}\eta \; \end{array}$$

(58b) $$\begin{array}{cc} & \;\;\;\;\;\;\;\;\;\;s.t.\sum_{n=1}^{N}\sum_{k=1}^{K}a_{k}\left[n\right]{\bar{R}}_{k}\left[n\right]\geq \eta ,\; \end{array}$$

(58c) $$\begin{array}{cc} & \;\;\;\;\;\;\;\;\;\;\;\;\;\;\;\;\;0\leq a_{k}\left[n\right]\leq 1,\forall k,n,\; \end{array}$$

  (20h),(20i).

With the relaxation, the optimal solution to (58) is an upper bound of (21). Now, problem (58) is a standard linear programming (LP) problem, which can be solved via CVX [39].

### 3.3. Transmit Power Optimization with Given Trajectory and Scheduling

The energy of IoT devices is usually limited, therefore, it is very necessary to optimize the transmit power. For given trajectory Q and scheduling A, problem (21) can be represented as

(59a) $$\begin{array}{cc} & \;\;\;\;\;\;\;\;\;\;\;\;\;\;\;\;\;\max_{\left\{P,\eta \right\}}\eta \; \end{array}$$

  s.t.(20f),(20g),(20b).

Equation (59) is a normative convex optimization problem. It can be solved effectively by CVX [39].

### 3.4. Overall Algorithm

In summary, by iteratively solving three convex optimization subproblems, the original problem (22) can be effectively solved. Algorithm 1 gives the proposed algorithm. Moreover, the convergence verification and complexity analysis will be elaborated as follows.
**Algorithm 1** Robust iterative algorithm for Problem (21)1:**Initialization:** Initialize feasible solution $\left(Q^{\left(i\right)},A^{\left(i\right)},P^{\left(i\right)}\right)$, iteration number i=0, and accuracy tolerance ε.2:**Repeat**3:   Give $P^{\left(i\right)}$ and $A^{\left(i\right)}$, then solve the convex optimization problem (56) and obtain the optimal solution $Q^{\left(i+1\right)}$.4:   Update optimization variables. Give $Q^{\left(i+1\right)}$ and $P^{\left(i\right)}$ and solve the convex optimization problem in (58) and obtain the optimal solution $A^{\left(i+1\right)}$.5:   Update optimization variables. Give $Q^{\left(i+1\right)}$ and $A^{\left(i+1\right)}$ and solve the convex optimization problem in (59) and obtain the optimal solution $P^{\left(i+1\right)}$.6:   Update i=i+1.7:**Until** the objective function’s value increases below the accurate tolerance ε.

Complexity analysis of Algorithm 1: The computational complexity can be concluded as $O\left(\Theta^{3.5}\right)\log\left(\frac{1}{\varepsilon }\right)$ [37], which is the complexity of the interior-point method, and where Θ is the number of variables, and ε is the accurate tolerance. Assuming that the number of iterations is *O*, thus, the computational cost of Algorithm 1 is estimated to be

(60) $$O\left(O{\left(\left(13+11K\right)N\right)}^{3.5}\right)\log\left(\frac{1}{\varepsilon }\right),$$

which means that the algorithm can be efficiently solved with a polynomial time complexity.

Convergence Verification: Constraint (20f) in problem (20) guarantees the optimal solution has an upper bound. In addition, it can be demonstrated that the lower bound will not decrease in each iteration [31], which verify the convergence of Algorithm 1.

## 4. Simulations and Discussion

### 4.1. Simulation Setup

In this section, we provide simulation results to validate the effectiveness of our proposed joint robust optimization algorithm under the PL channel model (denoted by “PL”).

Unless otherwise stated, the required simulation parameters are set as follows [35]. Assume that the UAV flies from the starting point $q^{start}=\left(100,200,100\right)$ to the ending point $q^{end}=\left(350,-200,150\right)$ within T=30 s, and collects information from K=4 IoT devices on the ground. The positions of K1 to K4 are set as $\left(150,100,0\right)$, $\left(200,50,0\right)$, $\left(250,0,0\right)$, and $\left(225,-100,0\right)$, respectively. The estimated position of the jammer is $\left(150,100,0\right)$ with $Q_{j}=30$. The channel model parameters are set as b=10, c=0.6. Other parameters are listed in Table 1.

The initial trajectory is that the UAV flies in a straight line from the starting point to the ending point, and the IoT devices are scheduled with the same number of time slots in sequence during the mission time period. Simultaneously, IoT devices transmit information with the average power of $\bar{p}$ when scheduled. In order to verify the proposed algorithm, we compared the experimental data with the following schemes:

(1) “SPL” scheme, i.e., anti-jamming robust trajectory and resource optimization under the simplified probabilistic LoS model. The channel power gain from UAV to the *k*th device is

(61) $$\beta_{k}^{SPL}\left[n\right]=\left\{\begin{array}{c} \beta_{0}d_{k}^{-\alpha }\left[n\right],\\ \mu \beta_{0}d_{k}^{-\alpha }\left[n\right], \end{array}\right.\mu <1,$$

where α represents the U2G path loss index. The possibility of LoS linking depends on the relative position and the elevation angle between the UAV and the IoT devices. We can express the LoS and NLoS probability with the following equations:

(62) $$P_{L}^{SPL}\left(\theta \right)=\frac{1}{1+be^{\left(-c\left(\theta -b\right)\right)}},$$

(63) $$P_{N}^{SPL}\left(\theta \right)=1-P_{L}^{SPL}\left(\theta \right),$$

where b>0 and c>0 are constants determined by the practical environment, and θ is the elevation angle between the UAV and the IoT device *k*.

Therefore, the channel power gain between the UAV and the *k*th IoT device can be given as

(64) $$\begin{array}{c} {\bar{g}}_{k}\left[n\right]\overset{\Delta }{\;=}E\left[{\left|g\right|}^{2}\right]=\\ P_{L}^{SPL}\left(\theta \right)\beta_{0}^{SPL}d_{k}^{-\alpha }\left[n\right]+\left(1-P_{L}^{SPL}\left(\theta \right)\right)\partial \beta_{0}^{SPL}d_{k}^{-\alpha }\left[n\right]\\ =\left[P_{L}^{SPL}\left(\theta \right)+\left(1-P_{L}^{SPL}\left(\theta \right)\right)\partial \right]\beta_{0}^{SPL}d_{k}^{-\alpha }\left[n\right]\\ \overset{\Delta }{\;=}{\bar{P}}_{L}^{SPL}\left(\theta \right)\beta_{0}^{SPL}d_{k}^{-\alpha }\left[n\right], \end{array}$$

where α<1 is the additional signal attenuation factor under the NLoS link.

The data rate at the *k*th IoT device during the time *T* can be expressed as

(65) $$R_{k}^{SPL}\left[n\right]=\log_{2}\left(1+\frac{p_{k}\left[n\right]{\bar{g}}_{k}\left[n\right]}{\sigma^{2}+{\bar{g}}_{j}\left[n\right]p_{j}}\right).$$

(2) “OnlyTra.” scheme is an algorithm that only optimizes the UAV trajectory without optimizing the IoT devices’ transmit power and scheduling.

(3) “PL-Nonrobust” scheme, which is the special case of the “robust optimization" assuming $Q_{j}=0$.

### 4.2. Performance Comparison

For the convenience of explanation, we focus on comparing the PL and SPL schemes and evaluating the influence of multiple parameters on UAV trajectory design and throughput performance.

**Figure 2:**
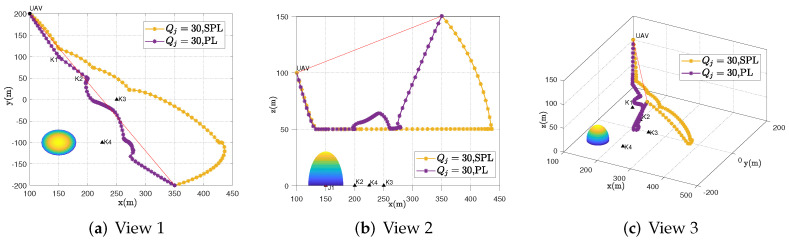
UAV’s behavior in the case of $Q_{j}=30$ in different views.

Figure 2 shows the UAV trajectory under the settings of $Q_{j}=30$ of different algorithms in T=30 s. The following phenomena can be observed: it can be seen that compared with the SPL scheme, the UAV in the PL scheme will be closer to the scheduled IoT devices in the horizontal position, and the flight altitude of the UAV will be different from the SPL scheme. The reason is that the UAV in the PL scheme will increase the elevation angle with the IoT devices as much as possible to obtain the angle gain further. In addition, it is worth noting that adjusting the UAV to a higher altitude will increase the elevation angle but at the same time will cause greater path loss. Hence, there is an optimal balance between elevation angle and distance. In the UAV 3D trajectory design that we considered, the UAV altitude should be optimized to balance the trade-off between path loss and attenuation (or distance and angle) to obtain higher throughput. In the SPL model, the trade-off between elevation angle and distance cannot be reflected. In summary, the UAV communication system based on the probabilistic LoS channel model can make full use of the advantages of the angle and distance trade-off to achieve more accurate trajectory design.

**Figure 3:**
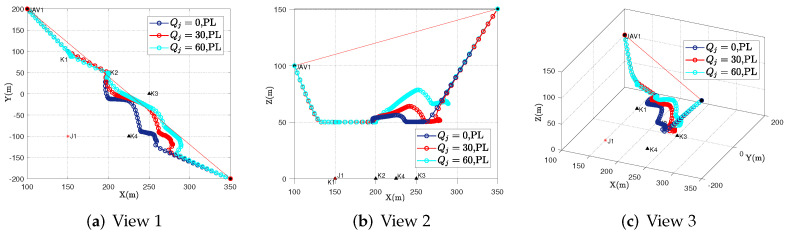
UAV’s behavior in the cases of $Q_{j}=0$, $Q_{j}=30$, $Q_{j}=60$ in different views.

Figure 3 shows the trajectory of the UAV when faced with different jamming ranges, for instance, $Q_{j}=0$, $Q_{j}=30$, and $Q_{j}=60$. It can be seen that with the increase of uncertainty radius, the UAV will adjust its horizontal position and altitude to move further away from the jammer to increase system throughput. The reasons are as follows. On the one hand, within the uncertainty range, jamming signal is assumed, which is the lower bounded of our proposed robust iterative algorithm in practice. Furthermore, with the increase in uncertainty of the jammer’s location, the jamming signal generates a greater threat. On the other hand, when $Q_{j}=0$, i.e., the non-robust scenario, it is the upper-bound in all cases. In a word, the proposed algorithm in this paper can effectively solve the reliable communication of UAV under a malicious jamming attack with an uncertain location. The UAV’s trajectory in Figure 3 meets our expectations, which verifies the effectiveness of our proposed algorithm.

**Figure 4:**
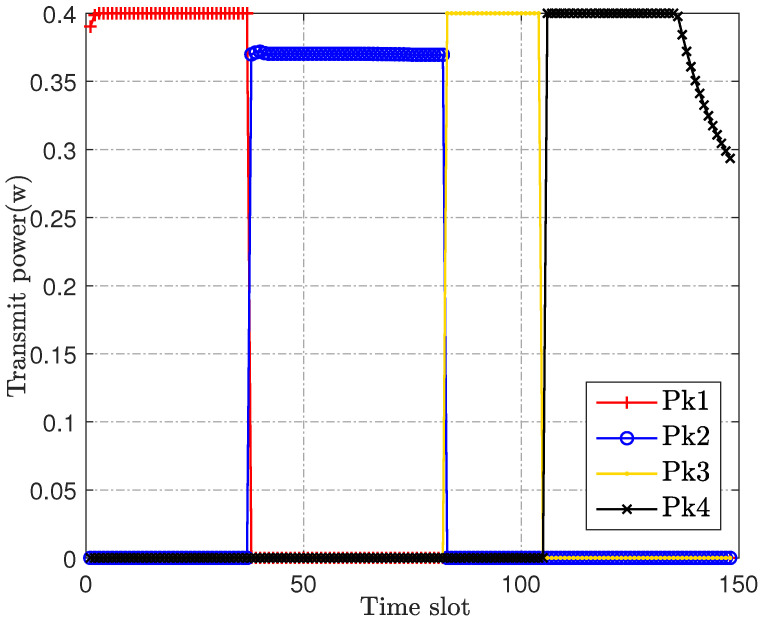
IoT devices’ transmit power.

**Figure 5:**
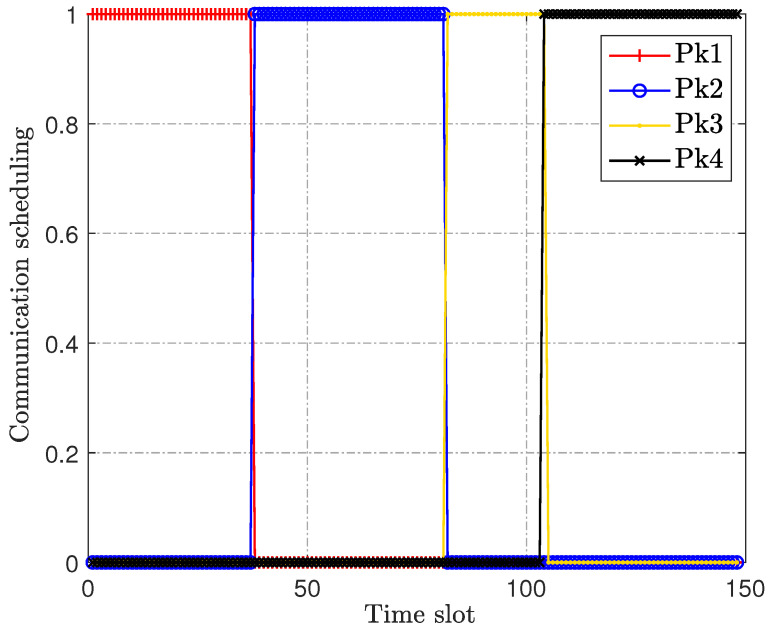
IoT devices’ transmit scheduling.

Figure 4 and Figure 5 are the optimization of the IoT devices’ transmit power and scheduling, respectively. Combined with the trajectory in Figure 2, it can be observed that the UAV will associate the IoT devices flexibly according to the actual location of the IoT devices. In addition, the IoT devices’ transmit power will be at or near the maximum for information transmission once they are associated with the UAV, for which an intermediate speed is not the optimal choice for our optimization objective, nor can it achieve the best anti-jamming effect. Therefore, Figure 4 and Figure 5 verify the effectiveness of the transmit power allocation and scheduling design of our proposed joint robust iterative optimization.

**Figure 6:**
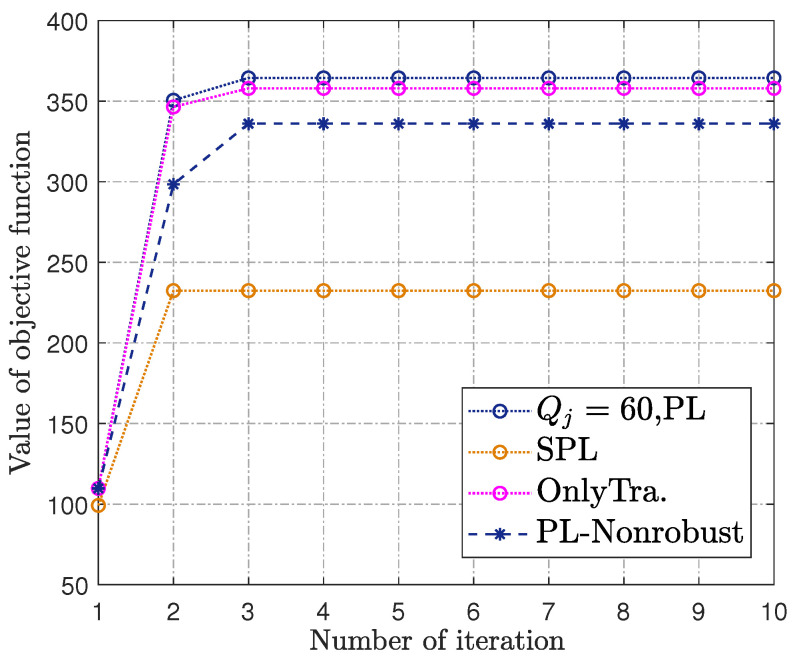
The convergence behaviors.

The convergence behaviors of our proposed algorithm are as shown in Figure 6. It can be seen that the throughput based on the **PL** channel model increases monotonically during the flight duration and quickly converges after iterations within five times, as only convex optimization problems need to be solved in each iteration of Algorithm 1, i.e., Step 3, Step 4, and Step 5. Moreover, all of them are of polynomial complexity. Therefore, the BCD method converges quickly for the setup of a moderate number of IoT devices, which verifies the effectiveness of Algorithm 1.

**Figure 7:**
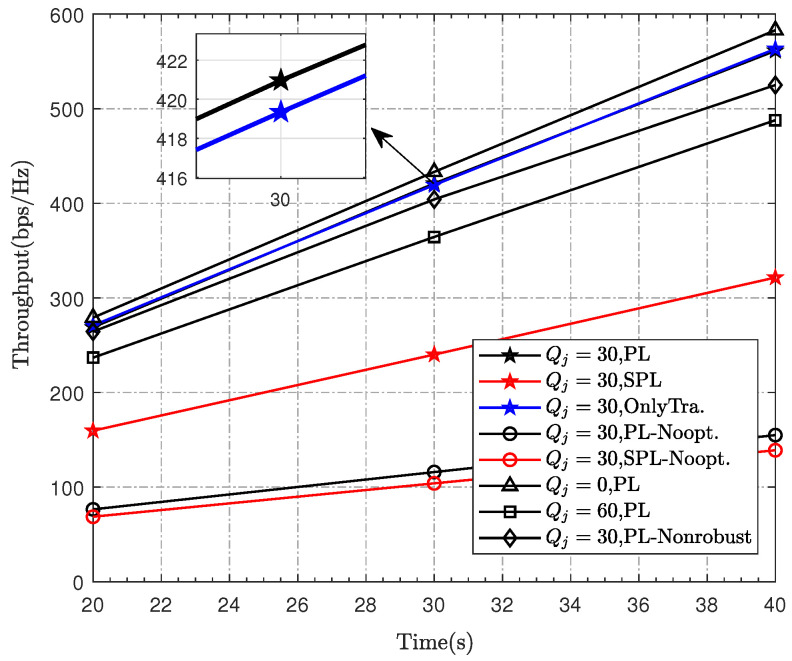
The maximum throughput versus time.

Figure 7 shows the overall throughput of the IoT devices’ derived by different algorithms (“PL”, “SPL”, “OnlyTra.”, “PL-Nonrobust”) under different *T* and depicts the impact of the uncertain position of the jammer for different cases. The following phenomena can be observed: (i) First, it can be observed that the throughput is improved as the UAV flight time increases in all cases. (ii) Compared with the schemes of “PL-30-Noopt” and “SPL-30-Noopt” (i.e., the original algorithm), our proposed optimization algorithm significantly improved the throughput, which indicates the effectiveness of our proposed algorithm. Moreover, the performance of joint optimization is better than that of only trajectory optimization. (iii) In addition, under the same settings, the PL scheme is superior to the SPL scheme. This is because PL makes good use of the accurate angle-dependent channel model. (iv) Further, we simulate the throughput under the PL-Nonrobust algorithm for comparison. It can be found that the robust algorithm can greatly improve the throughput, which reflects the superiority of our proposed robust algorithm. (v) By observing the throughput under different radius settings, it can be seen that as the uncertainty radius increases, the throughput decreases, which indicates the effectiveness of the proposed robust algorithm. In summary, by adopting a practical probabilistic channel model and performing robust joint optimization, the system performance has been dramatically improved.

## 5. Conclusions

This paper studied the anti-jamming trajectory and resource optimization of a UAV-enabled IoT system in the presence of a jammer with imperfect location information. The UAV 3D trajectory, IoT devices’ transmit power, and scheduling were jointly optimized to maximize the throughput based on the probabilistic LoS channel model. First, we established the probabilistic LoS channel model. Then, based on this model, we addressed the original optimization problem by leveraging the BCD method, SCA technology, D.C. programming approach, and S-procedure. Finally, by comparing with multiple benchmark schemes, the significant throughput performance improvement was shown in the numerical results, verifying the effectiveness of considering the more accurate probabilistic LoS channel model in anti-jamming 3D trajectory and resource optimization for UAV-enabled IoT networks.

## Figures and Tables

**Table 1:** Simulation parameters.

Notation	Physical Meaning	Value
$H_{\min}$	UAV minimum flight altitude	50 m
$H_{\max}$	UAV maximum flight altitude	150 m
$V_{\max}$	UAV maximum flight speed	50 m/s
Δt	Time slot	0.2 s
$Q_{j}$	Radius of the hemisphere	30 m
$P_{j}$	Jammer’s transmit power	0.4 W
$\bar{p}$	The *K*’s average transmit power	0.1W
$p_{max}$	The *K*’s maximum transmit power	$4\bar{p}$
$\sigma^{2}$	The power of AWGN	−110 dBm/Hz
$\beta_{0}$	The path loss at $d_{0}=1$ m	−40 dB
ε	The accuracy tolerance	$10^{-3}$
$\alpha_{L}$	The LoS path loss exponent	2.2
$\alpha_{N}$	The NLoS path loss exponent	3.2
μ	Additional signal attenuation factor	−20 dB

## Data Availability

Data available based upon reasonable request from corresponding author.

## Abbreviations

The following abbreviations are used in this manuscript:

UAV	unmanned aerial vehicle
IoT	Internet of Things
LoS	line-of-sight
BCD	block coordinate descent
SCA	successive convex approximation
D.C	difference of convex
3D	three-dimensional
U2G	UAV to ground
NLoS	non-line-of-sight
PL	probabilistic LoS
AWGN	additive white Gaussian noise
LP	linear programming
